# Activation of invariant natural killer T cells stimulated with microbial α-mannosyl glycolipids

**DOI:** 10.1038/s41598-017-10309-x

**Published:** 2017-08-29

**Authors:** Michio Shimamura, Masaki Yamamura, Tatsuya Nabeshima, Naoki Kitano, Peter van den Elzen, Hasan Yesilkaya, Peter Andrew, Petr Illarionov

**Affiliations:** 1 Tsukuba Research Center for Interdisciplinary Materials Science, University of Tsukuba, Tsukuba, 305-8571 Japan; 2 Graduate School of Pure and Applied Sciences, University of Tsukuba, Tsukuba, 305-8571 Japan; 3 Department of Health and Dietetics, Faculty of Health and Medical Science, Teikyo Heisei University, Higashi-Ikebukuro, Tokyo, 170-8445 Japan; 4 Mitsubishi Kagaku Institute of Life Sciences, Tokyo, 194-8511 Japan; 5 Child and Family Research Institute, University of British Columbia, Vancouver, V5Z4H4 BC Canada; 6 Department of Infection, Immunity and Inflammation, University of Leicester, Leicester, LE1 9HN United Kingdom; 7 School of Biosciences, University of Birmingham, Birmingham, B15 2TT United Kingdom

## Abstract

Some synthetic and bacterial glycolipids presented by CD1d specifically activate invariant NKT (iNKT) cells bearing an invariant Vα14-Jα18 (mouse) or Vα24-Jα18 (human) TCR. The antigenic glycolipids identified to date consist of two hydrophobic chains and an α-glycoside in which the 2′-OH group is in the *cis* orientation toward the anomeric group, namely, either an α-galactoside or an α-glucoside. Several microbial α-mannosyl glycolipids, in which the 2′-OH group is in the *trans* orientation, were herein examined to establish whether they have potential to activate iNKT cells. We found that α-mannnosyl1-3 (6′-O-acyl α-mannosyl)-1-1 monoacylglycerol and cholesteryl 6′-O-acyl α-mannoside, found in *Saccharopolyspora* and *Candida albicans*, respectively, induced the activation of iNKT cells, dependent on CD1d. In contrast, α-mannosyldiacylglycerol found in *Streptococcus suis* or α-mannosylceramide demonstrated markedly less antigenicity for iNKT cells. The potentially antigenic α-mannosyl glycolipids contributed to the protection of mice against infection with *S*. *pneumoniae* in which iNKT cells have previously been found to participate. Furthermore, these glycolipids induced the production of proinflammatory cytokines by macrophages, thereby suggesting their recognition by specific pattern recognition receptors (PRRs). Collectively, these results suggest that these microbial α-mannosyl glycolipids are capable of being recognized by both the invariant TCR and PRRs and inducing immune responses.

## Introduction

Invariant NKT (iNKT) cells bearing invariant Vα14-Jα18 (mouse) or Vα24-Jα18 (human) TCR (type I NKT cells) are a subset of T lymphocytes^1^ that are specifically activated with certain glycolipids presented by CD1d such as α-galactosyl ceramide (α-GalCer)^2^ isolated from marine sponges^3^. Some bacterial glycosphingolipids that are stereochemically similar to α-GalCer are also recognized by iNKT cells in a CD1-dependent manner, for example, α-glucuronosyl from *Sphingomonas* ^4^, α-galacturonosyl ceramides from α-proteobacteria^5^, α-galactosyl diacylglycerol from *Borrelia burgdorferi* ^6^ and α-glucosyl diacylglycerol from *Streptococcus pneumoniae* ^7^. (Refer to a recent review for antigens for iNKT cells^8^) These antigenic glycolipids possess an α-glycosyl moiety in which the 2′-hydroxyl group is equatorial and in the *cis* orientation toward the anomeric group. The importance of this equatorial 2′-hydroxyl group for the antigenicity of glycolipids was demonstrated by reduction in activity following the 2′-hydroxyl group substitution^9^.

We have recently demonstrated that cholesteryl 6′-O-acyl α-glucoside (ChAcGlc) induces immune responses from iNKT cells in a CD1d-dependent manner^10^. This finding suggests that even the cholesteryl residue is able to anchor to either the A′ or the F′ pocket of CD1d and that the α-glucose residue is recognized by the invariant TCR.

The natural occurrence of several α-mannosyl glycolipids has been reported to date. In the present study, we characterized some bacterial, fungal, and related α-mannosyl glycolipids to extend the criteria for the structural features of antigenic glycolipids for iNKT cells. We found that α-mannnosyl1-3 (6-acyl α-mannosyl)-1-1 monoacylglycerol (M-AcM-MAG) (formally 1-O-acyl-3-O-(α-mannosyl-(1–3)-α-(6′-O-acyl-mannosyl)-sn-glycerol) and cholesteryl 6′-O-acyl α-mannoside (ChAcMan) activated mouse and human iNKT cells in a CD1d-dependent manner. M-AcM-MAG was found in *Saccharopolyspora* ^11^ responsible for farmer’s lung disease^12^, *Rothia dentocariosa* ^13^ associated with infective endocarditis^14^ and *Arthrobacter* ^15^, whereas the occurrence of ChAcMan has been suggested in a mycelial form *Candida albicans* ^16, 17^. We also demonstrated that these α-mannosyl glycolipids induced proinflammatory immune responses from bone marrow-derived macrophages, suggesting that they were recognized by certain pattern recognition receptors (PRRs) for pathogen-associated molecular patterns (PAMPs) such as C-type lectin receptors^18^ as well as by iNKT cell receptors. Our results raise a possibility that a potential cross-priming of the invariant TCR and any innate immune receptors with the microbial α-mannosyl glycolipids allows the host to effectively amplify the immune responses to infection.

## Results

### Microbial α-mannosyl glycolipids stimulate invariant NKT cells *in vitro*

**Figure 1:**
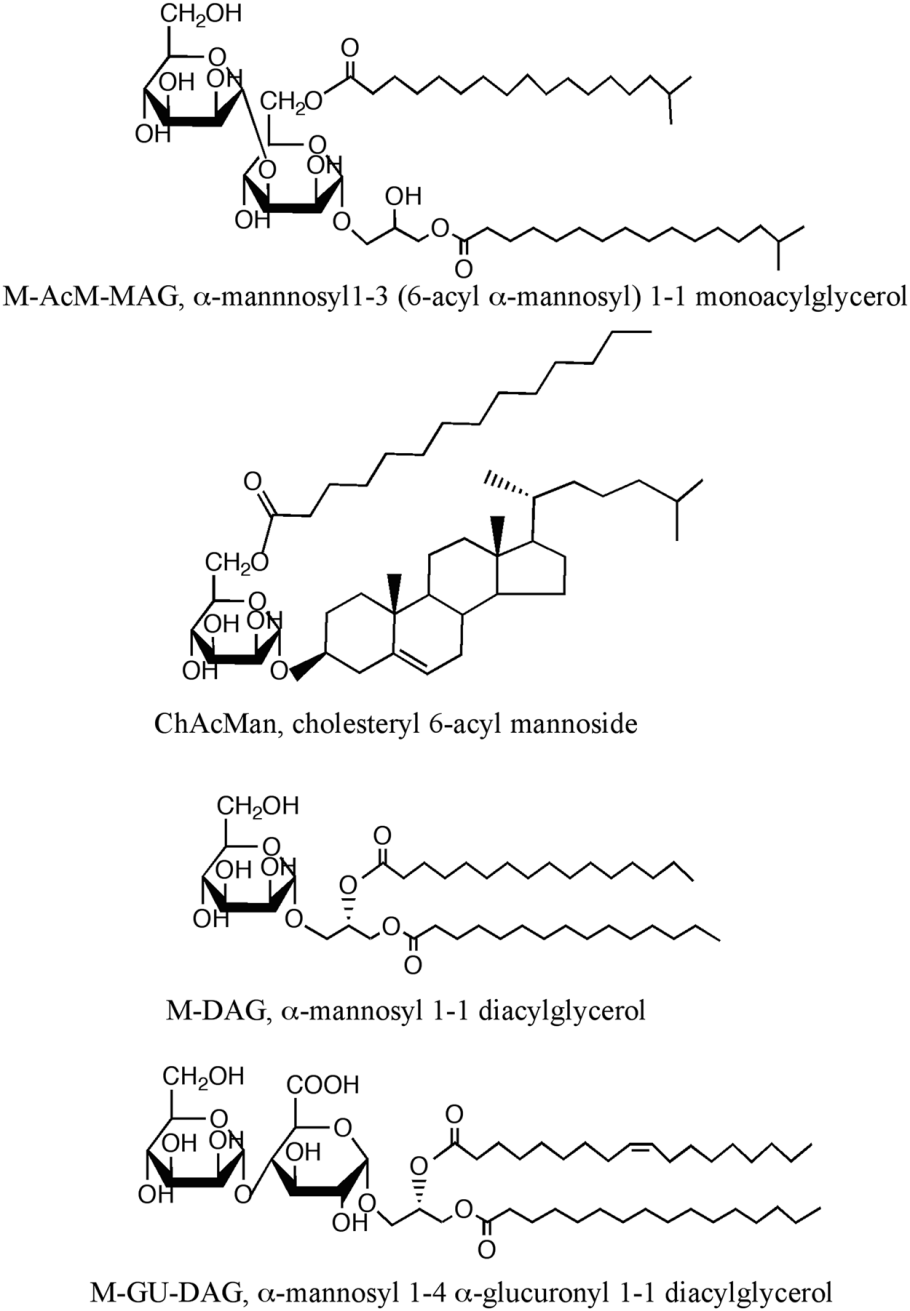
Structures of glycolipids evaluated in this study.

Accumulating evidence on the structural characterization of the glycolipid ligands for the TCR of iNKT cells suggests the preference of α-galactosyl or α-glucosyl glycolipids. In order to extend the knowledge on the characteristics of antigens that are able to stimulate iNKT cells, several bacterial and fungal α-mannosyl glycolipids listed in Fig. 1, and others, were tested in the present study to determine whether they are stimulative for iNKT cells.

**Figure 2:**
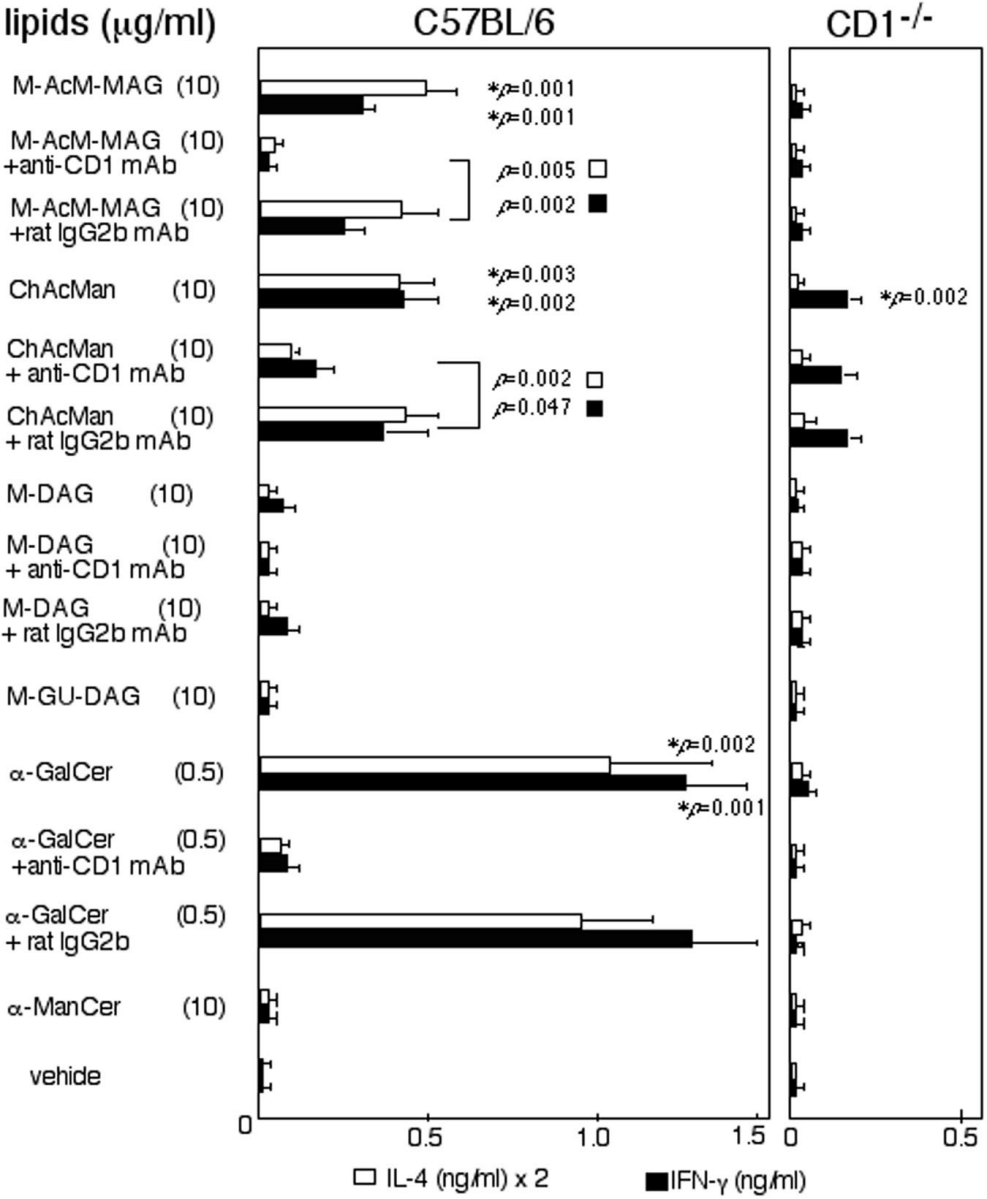
Stimulation with microbial α-mannosyl glycolipids induced immune responses by invariant NKT cells. Liver MNCs prepared from C57BL/6 and CD1^−/−^ mice were cultured in the presence of the microbial α-mannosyl glycolipids, α-GalCer or α-ManCer. Anti-CD1 or control antibodies were added to some cultures. Cytokine concentrations in the culture supernatants after 2 days were measured by ELISA. The mean values of the triplicate cultures are shown. Experiments were repeated 6 times, and similar results were obtained. *P* values in the Student’s *t*-test are shown in the figure. *p indicates the values relative to the control cultures (with vehicle), while *p* demonstrates those in a comparison between the cultures with anti-CD1 antibodies and with the isotype matched control antibodies. In the comparison of the cytokine production by C57BL/6 and CD1^−/−^ liver MNCs in the culture with M-AcM-MAG (10 μg/ml) or ChAcMan (10 μg/ml), the *p* values were less than 0.01 (IL-4) or 0.05 (IFN-γ) in the Student’t *t*-test.

Mononuclear cells (MNCs) were prepared from the livers of C57BL/6 or CD1^−/−^ mice, and they were cultured in the presence of α-mannosyl glycolipids. The immune responses of these cells were assessed by measuring their production of cytokines (Fig. 2). MNCs from C57BL/6 mice were induced to produce both IL-4 and IFN-γ in the presence of M-AcM-MAG and ChAcMan, while these cells secreted lower amounts of cytokines with other α-mannosyl glycolipids such as α-mannosyl diacylglycerol (M-DAG) found in Streptococcus suis^7^, α-ManCer and α-mannosyl 1–4 α-glucuronyl 1-1 diacylglycerol (M-GU-DAG) found in *Corynebacterium glutamicum* ^19^. The immune responses of C57BL/6 MNCs to M-AcM-MAG and ChAcMan were significantly reduced in the presence of anti-CD1 blocking antibodies (Fig. 2, p < 0.05). Furthermore, the immune responses of CD1^−/−^ liver MNCs to these glycolipids were markedly weaker than those of the wild-type responders. The dose-dependency of the immune responses by C57BL/6 and CD1^−/−^ liver MNCs are also shown in the Supplemental Information (Figure S1). These results indicate that the immune responses detected in the culture of C57BL/6 liver MNCs were CD1-dependent. It is important to note that the blockade of ChAcMan-induced IFN-γ production, but not IL-4 production, by C57BL/6 cells with anti-CD1 antibodies was only partial and that the substantial production of IFN-γ by CD1^−/−^ responders was observed. Thus, these results demonstrate that immune responses to ChAcMan but not to M-AcM-MAG are in part independent of CD1d restriction.

**Figure 3:**
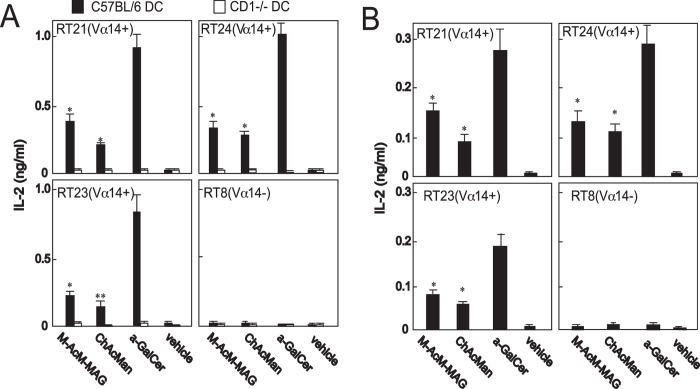
Activation of invariant NKT cell hybridomas with microbial α-mannosyl glycolipids. (**A**) Stimulation with DCs loaded with glycolipids. DCs were prepared from the bone marrow of C57BL/6 and CD1^−/−^ mice. They were incubated for 16 h in the presence of the indicated glycolipids. Hybridomas derived from either invariant Vα14 cells (RT21, RT23 and RT24) or an irrelevant Vβ8^+^ T cell (RT8) (used as a control) were cultured for 1 day with the lipid-loaded DCs. The secretion of IL-2 into culture supernatants was measured by ELISA. Mean values of the triplicate cultures are shown. Experiments were repeated for 3 times, and similar results were obtained. *p < 0.005; **p < 0.020 in the Student’s *t*-test (relative to the control cultures with DCs loaded with the vehicle (DMSO)). (**B**) Stimulation with immobilized recombinant CD1d/β2 m proteins loaded with glycolipids. Plastic wells were pre-coated with CD1d/β2 m dimers, and hybridomas (RT24, RT23, RT21 or RT8) were cultured for 1 day on the wells. The secretion of IL-2 into culture supernatants was measured by ELISA. The mean values of triplicate cultures are shown. Experiments were repeated for twice, and essentially similar results were obtained. *p < 0.01 in the Student’s *t*-test (relative to the control cultures loaded with the vehicle (DMSO)).

Immune responses to the α-mannosyl glycolipids by iNKT cell hybridomas were examined to make sure that one of the responder cells among C57BL/6 liver MNCs were iNKT cells (Fig. 3A). Dendritic cells (DCs) prepared from the bone marrow cells of C57BL/6 or CD1^−/−^ mice were loaded with glycolipids, and these cells were co-cultured with the hybridomas derived from either invariant Vα14 TCR^+^ NKT cells or an irrelevant Vβ8^+^ T cell^20^. The immune responses of these cells were monitored by analyzing their production of IL-2. The iNKT cell hybrid lines (RT21, RT23, RT24) were stimulated by C57BL/6 DCs previously loaded with M-AcM-MAG or ChAcMan as well as with α-GalCer, whereas an irrelevant T cell hybridoma (RT8) was not. The stimulation of iNKT cell hybridomas with these α-mannosyl glycoplipids was not observed when these cells were stimulated with CD1^−/−^ DCs. The activation of iNKT cell hybridomas with M-AcM-MAG and ChAcMan was similarly demonstrated when these cells were stimulated with the glycolipids presented by the CD1d proteins immobilized on the plate prior to the culture (Fig. 3B).

Collectively these results indicate that one of the principal populations among the liver MNCs responding to the α-mannosyl glycolipids was CD1-restricted iNKT cells.

### Staining of iNKT cells with fluorescence-labeled tetramers of the CD1d/α-mannosyl glycolipid complex

**Figure 4:**
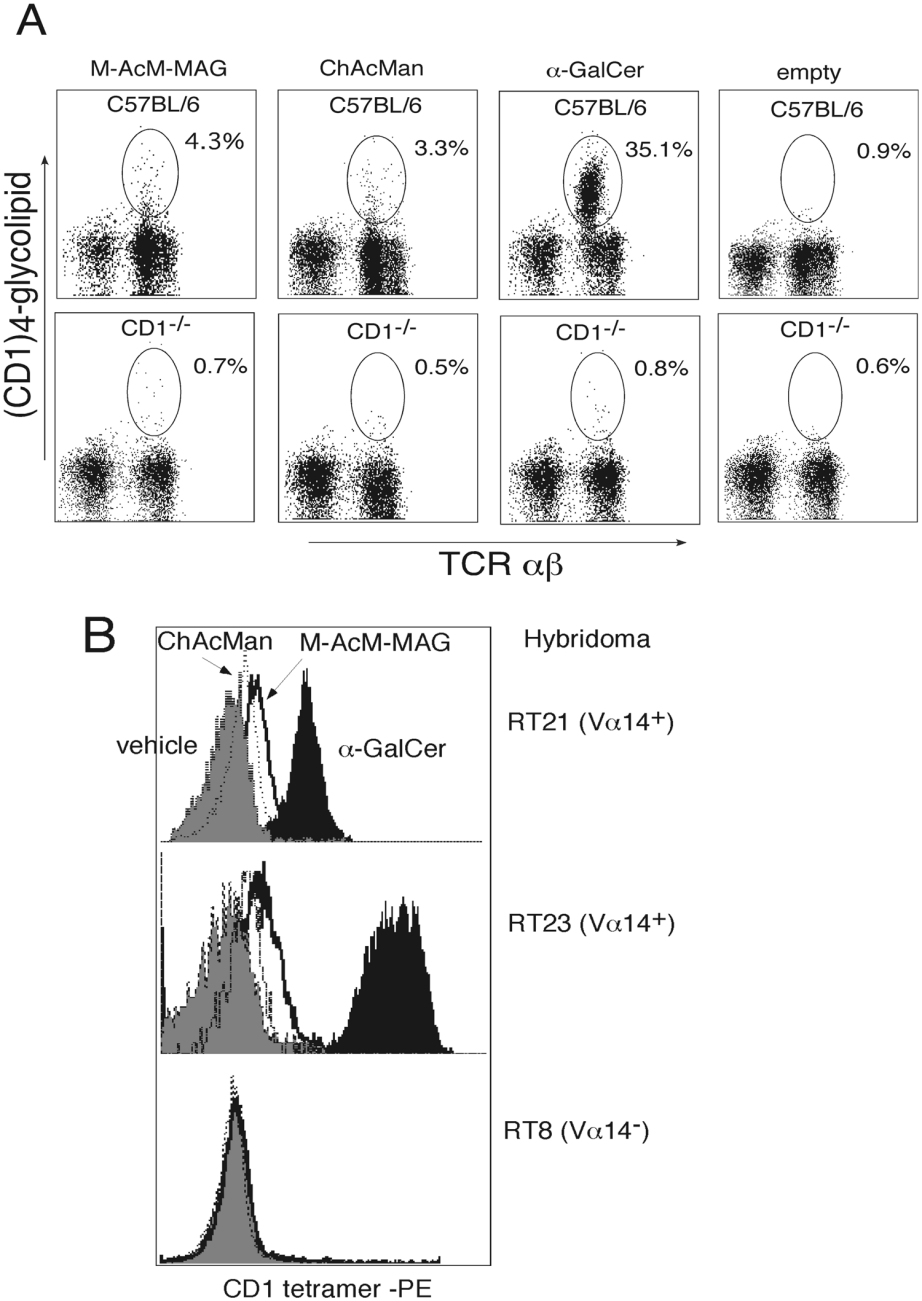
Staining of iNKT cells with fluorescence-labeled tetramers of the CD1/α-mannosyl glycolipid complex. (**A**) Liver MNCs from C57BL/6 or CD1^−/−^ mice were stained with PE-labeled tetramers of the CD1/glycolipid complex accompanied with anti-TCR Cβ mAb-FITC, and analyzed by flow cytometry. Samples were gated on the lymphoid cells in the forward versus side scatter pattern. A substantial fraction of liver MNCs from C57BL/6 but not CD1^−/−^ mice was stained with the CD1/M-AcM-MAG or CD1/ ChAcMan complex. (**B**) Hybridomas derived from either invariant Vα14 cells (RT21, RT23) or an irrelevant Vβ8^+^ T cell (RT8) were stained with the PE-labeled tetramers of CD1 coupled with M-AcM-MAG, ChAcMan or α-GalCer, and analyzed by flow cytometry. RT21 and RT23 but not RT8 were substantially stained with the CD1/α-mannosyl glycolipid complex.

Liver MNCs from C57BL/6 or CD1^−/−^ mice were stained with the CD1d tetramers previously loaded with the α-mannosyl glycolipids and analyzed by flow cytometry to confirm the CD1-restricted recognition of these glycolipids by iNKT cells (Fig. 4A). A substantial fraction of liver MNCs isolated from C57BL/6 was stained with the CD1/M-AcM-MAG complex (4.3%) and the CD1/ ChAcMan complex (3.3%), whereas much smaller fractions of CD1^−/−^ liver cells were stained by these complexes. Cell staining with the α-mannosyl lipid and CD1 complex was glycolipid-dependent because markedly smaller fractions of cells were stained with the empty CD1 tetramers (0.9% in the wild type liver MNCs). These results suggest that M-AcM-MAG and ChAcMan presented by CD1 are recognized by a fraction of iNKT cells.

The cells of hybrid lines derived from either invariant Vα14 cells (RT23, RT21) or an irrelevant Vβ8^+^ T cell (RT8) were then stained with fluorescence-labeled CD1 tetramers loaded with M-AcM-MAG, ChAcMan or α-GalCer and analyzed by flow cytometry (Fig. 4B). The hybridomas derived from iNKT cells were stained with a complex of CD1d tetramers and M-AcM-MAG or ChAcMan to a certain extent, although the intensity of the fluorescence staining was weaker than when iNKT cell hybribomas were stained with the tetramers of a CD1/α-GalCer complex as shown in the liver iNKT cells (Fig. 4A). On the other hand, no hybrid cells derived from an irrelevant T cell were stained with the CD1d tetramers loaded with any glycolipids. Taken together, these results strongly suggest that specific α-mannosyl glycolipids in the context of CD1d are recognized by a fraction of invariant Vα14 TCR-bearing cells.

### Preferential activation of iNKT cells among liver MNCs following the stimulation with α-mannosyl glycolipids

**Figure 5:**
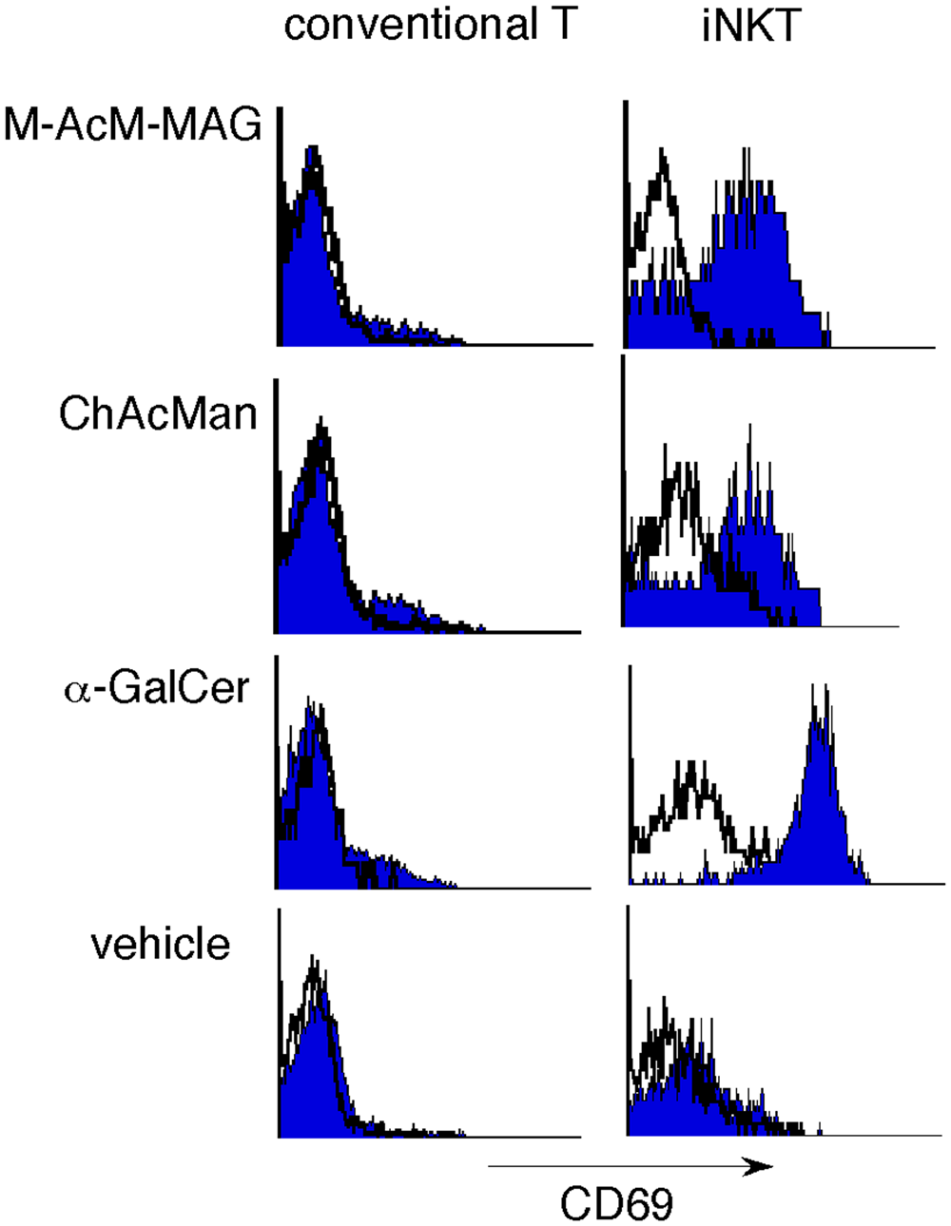
Preferential activation of iNKT cells among liver MNCs following stimulation with α-mannosyl glycolipids. Liver MNCs prepared from wild-type mice were cultured in the presence of α-mannosyl glycolipids. After two days cells were triply stained with fluorescence-labeled anti-CD69 antibodies, anti-TCR Cβ antibodies and CD1/α-GalCer tetramers, and CD69 expression in both iNKT cell (TCR Cβ^+^, CD1/α-GalCer tetramer^+^) and conventional T cell populations (TCR Cβ^+^, CD1/α-GalCer tetramer^−^) was analyzed by flow cytometry.

Liver MNCs were cultured in the presence of α-mannosyl glycolipids and the expression of the activation markers for T cells was examined to identify the cell populations responsible for the stimulation with the glycolipids. Cells after cultivation were triply stained with fluorescence-labeled anti-CD69 antibodies, anti-TCR Cβ antibodies and CD1d/α-GalCer tetramers and the expression of CD69 by iNKT and conventional T cell populations (TCRCβ^+^, CD1d/α-GalCer tetramers^+^ and TCRCβ^+^, CD1d/α-GalCer tetramers^−^, respectively) was analyzed (Fig. 5). It is clearly shown that CD69-positive cells were enriched in the iNKT cell population after the stimulation with M-AcM-MAG or ChAcMan. These results strongly suggest the preferential activation of iNKT but not conventional T cells in response to stimulation with these α-mannosyl glycolipids as well as with α-GalCer.

### Human invariant Vα24-Jα18 TCR-bearing cells respond to the stimulation with α-mannosyl glycolipids in a CD1d-dependent manner

**Figure 6:**
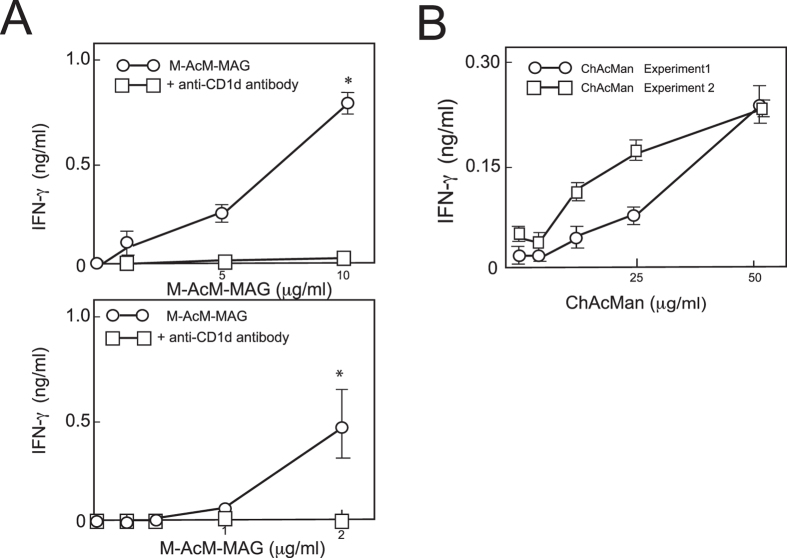
Human invariant Vα24-Jα18 TCR-bearing NKT cells responded to the stimulation with M-AcM-MAG in a CD1d-dependent manner. Cells of human Vα24 NKT cell clone (BM2a.3) were stimulated with CD1d-transfected K562 cells or plate-coated human CD1d proteins previously loaded with α-mannosyl lipids as described in the Materials and Methods, and the IFN-γ production by BM2a.3 cells were measured by ELISA. (**A**) BM2a.3 cells were stimulated with the CD1d-transfected K562 cells (upper panel) or the plate-coated CD1d proteins (lower panel) previously loaded with M-AcM-MAG. In some experiments cells were cultured in the presence of anti-CD1d antibodies. Each point represents the average of triplicate cultures. Experiments were repeated for three times and one of them is shown. *p < 0.01 in the Student’s *t*-test (relative to the cultures with anti-CD1d antibody). (**B**) BM2a.3 cells were cultured on the CD1d-coated plates previously loaded with ChAcMan, and the IFN-γ in the culture supernatants was measured. Each point represents the average of triplicate cultures. Experiments were repeated for twice. The *p* values in the comparison between the cultures at the dose of 50 μg/ml ChAcMan and those with vehicle were less than 0.01 in the Student’s *t*-test in both the experiment 1 and 2.

To clarify whether the recognition of α-mannosyl glycolipids by iNKT cells is similar in mice and humans, the responses of human Vα24 TCR^+^ cells to these glycolipids were examined. Culture wells were pre-coated with recombinant human CD1d/β2 m proteins, and the immobilized proteins were loaded with M-AcM-MAG or ChAcMan. The cells of a human invariant Vα24 TCR^+^ NKT cell clone were cultured in the wells, and their production of IFN-γ was assessed (Fig. 6). Human iNKT cells were stimulated with these glycolipids in the context of CD1d/β2 m in a dose-dependent manner. Similarly, human iNKT cells were activated by CD1d-transfected DCs previously loaded with M-AcM-MAG (Fig. 6A). The stimulation was blocked in the presence of anti-CD1d antibodies. These results strongly suggest that human invariant Vα24 TCR^+^ cells as well as mouse invariant Vα14 TCR^+^ cells are responsive to these microbial α-mannosyl glycolipids.

### Recognition of microbial α-mannosyl glycolipids by PRRs for PAMPs

As shown in Fig. 2, IFN-γ production by wild-type liver MNCs after the stimulation with ChAcMan was only partially blocked in the presence of anti-CD1 antibody and the production of IFN-γ, but not IL-4, was observed even in the culture of liver cells from CD1^−/−^ mice after ChAcMan stimulation. These results strongly suggest that this glycolipid is capable of being recognized by cells other than invariant TCR-bearing cells.

**Figure 7:**
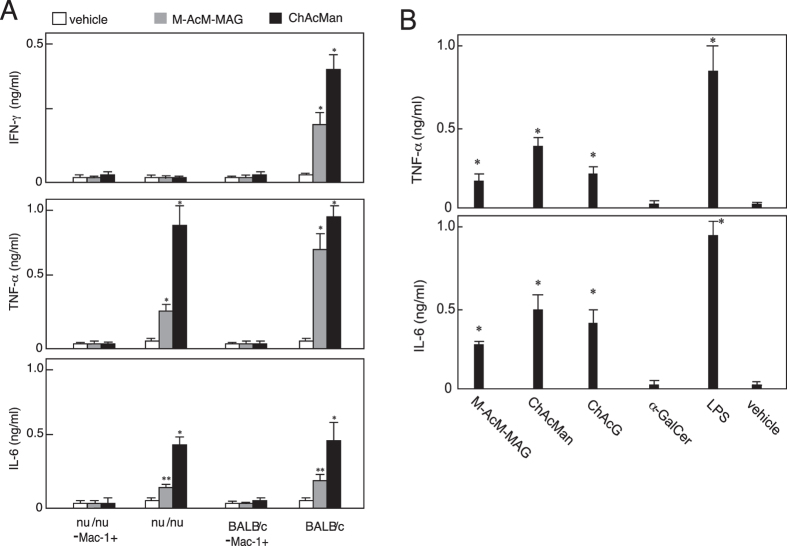
Certain α-mannosyl glycolipids are recognized by pattern recognition receptors of the innate immune system. (**A**) The MNCs of spleenocytes prepared from BALB/c and BALB/c (*nu/nu*) mice were treated with biotinylated anti-Mac-1 mAb followed by streptavidin-coated magnetic beads (Dynabeads, Veritas, Japan) to remove Mac-1^+^ cells. Splenocytes before and after the depletion of Mac-1^+^ cells were cultured in the presence of glycolipids. After 2 days, the concentrations of inflammatory cytokines were measured by ELISA. *p < 0.005, **p < 0.020 in Student’s *t*-test (relative to the control cultures in the presence of vehicle (DMSO)). (**B**) Bone marrow-derived macrophages prepared as described in the experimental section were cultured in the presence of glycolipids. After 2 days, the concentrations of inflammatory cytokines were measured by ELISA. *p < 0.01 in the Student’s *t*-test (relative to the control cultures in the presence of the vehicle (DMSO)).

Splenocytes prepared from *nu/nu* mice, lacking thymus-dependent T cells, as well as from wild-type mice, produced the inflammatory cytokines TNF-α and IL-6 in cultures in the presence of ChAcMan, and their production of these cytokines following the stimulation with ChAcMan was greater than that induced with M-AcM-MAG (Fig. 7A). Cytokine production was abolished when Mac-1^+^ cells were depleted from splenocytes for cultivation. Thus, Mac-1^+^ cells such as certain subsets of macrophages or DCs may play crucial roles as responders in the T cell-independent secretion of TNF-α and IL-6 in response to the ChAcMan stimulation. These cells may simultaneously have roles as antigen-presenting cells in triggering the production of IFN-γ by iNKT cells and by bystander T cell populations.

Macrophages were also prepared from bone marrow cells and their responses to the stimulation with these α-mannosyl glycolipids were examined. Those macrophages from wild-type mice produced TNF-α and IL-6 in the presence of the glycolipids in cultures as shown in Fig. 7B.

Taken together, these results suggest that α-mannosyl glycolipids, such as ChAcMan and M-AcM-MAG produced by microorganisms, are recognized not only by invariant Vα14 TCRs but also by certain innate receptors for microbial pathogens that are expressed by macrophages or DCs.

### *In vivo* evaluation of the activity of α-mannosyl glycolipids

**Figure 8:**
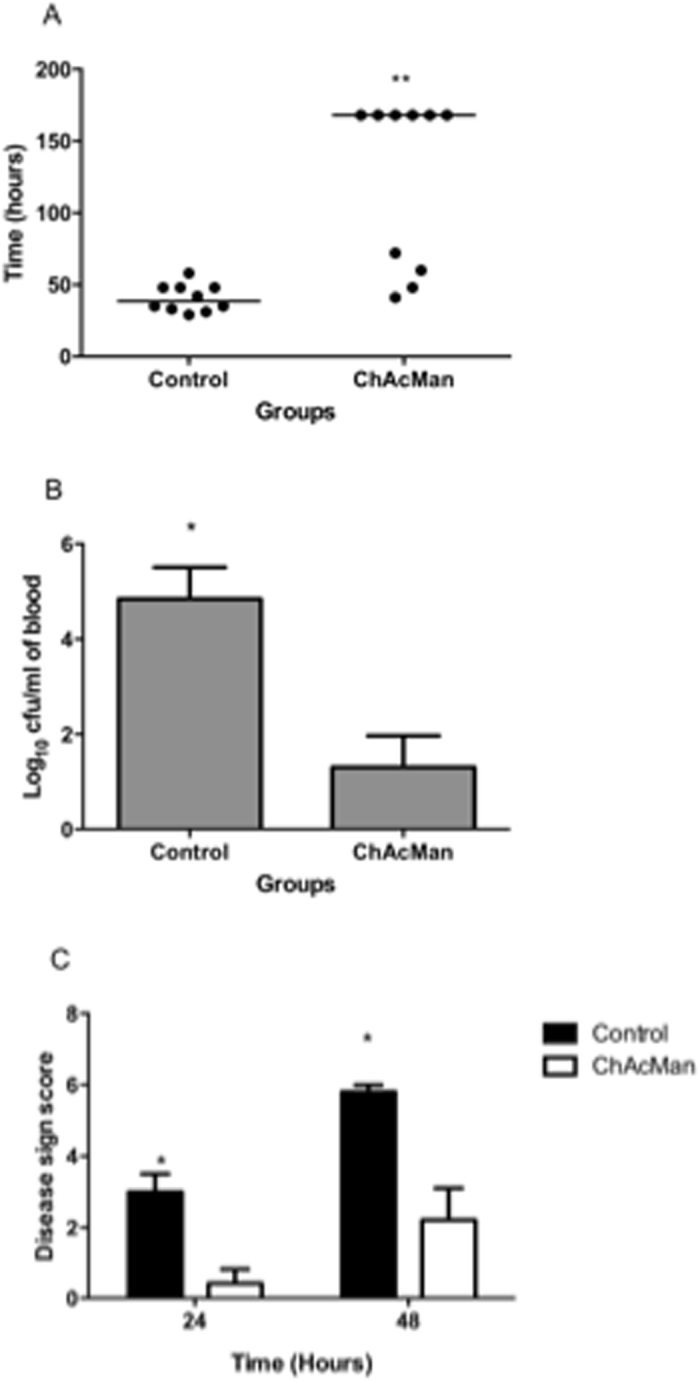
Prevention of *S*. *pneumoniae* infection *in vivo* by the administration with ChAcMan. (**A**) Mice were infected intranasally with *S*. *pneumonia*. ChAcMan was administered intraperitoneally 30 min post-infection. Disease progress in mice was evaluated as described in Materials and Methods. (**A**) The survival time. The mice administered with ChAcMan (median: 168 ± 58.8 h, n = 10) survived significantly longer than the control (injected with PBS) (median: 38.5 ± 9.5 h, n = 10) (***p < 0.01 in the Mann-Whitney U-test). (**B**) Bacterial counts in blood at 24 h post infection. ChAcMan administered mice (Log_10_ colony forming unit/ml: 1.3 ± 2.09, n = 10) had less bacteremia compared to the control (Log_10_ colony forming unit/ml: 4.84 ± 2.1, n = 10) (*p < 0.05 in the Bomferroni post test). (**C**) The signs of disease in the mice were quantified 24 and 48 h after infection. ChAcMan-administered mice were less severe (*p < 0.05 in the Mann-Whitney U-test for both time points).

Production of immunoregulatory cytokines, such as IL-4 and IFN-γ, was observed in the splenocytes isolated from wild-type but not CD1d^−/−^ mice that had been intravenously injected with ChAcMan or M-AcM-MAG 90 min before isolation (data not shown). It is likely that such CD1d-dependent prompt immune responses *in vivo* to glycolipid antigens are primarily responsible for iNKT cells. Since critical roles of iNKT cells in the protection of mice against *S*. *pneumoniae* infection have been reported^7, 21–23^, attempts were made to examine therapeutic potentials of these α-mannosyl glycolipids. Mice were infected intranasally with *S*. *pneumoniae* and then ChAcMan or M-AcM-MAG was injected intraperitoneally 30 min after infection. Mice were monitored for disease signs for 7 days (Fig. 8). The cohort injected with ChAcMan survived significantly longer than the control (*p* < 0.01, Fig. 8A). Moreover, mice administered with ChAcMan had less bacteria in blood than the control (Fig. 8B). Consistent with the survival time and blood counts, the signs of disease in mice administered with ChAcMan were less severe at 24 and 48 h post infection (Fig. 8C). Similar results were obtained from mice given ChAcMan intraperitoneally 12 h after infection (data not shown). Overall the findings strongly suggest that ChAcMan protects mice against lethal infection with *S*. *pneumoniae* by activation of iNKT cells. In contrast to ChAcMan, M-AcM-MAG was less effective in protecting mice from *S*. *pneumoniae* infection when 5 μg /mouse was administered intraperitoneally. Presumably a higher dose of this glycolipid is required to demonstrate the activity following intraperitoneal injection because of the susceptibility of acylglycerols to lipases or esterases.

## Discussion

In the present study, we showed that specific microbial α-mannosyl glycolipids (M-AcM-MAG and ChAcMan) have the potential to induce the activation of invariant NKT cells. We also demonstrated that these glycolipids, particularly ChAcMan, induced the secretion of inflammatory cytokines from macrophages and DCs, thereby suggesting that they are recognized by certain PRRs in innate immunity. In addition, we found that ChAcMan administration to mice protected them against infection with *S*. *pneumoniae*.

Glycolipids presented by CD1d and recognized by invariant TCRs have been shown to contain an α-galactosyl, an α-glucosyl residue or its substituent of the 3′- or 6′- hydroxyl group. On the other hand, no α-mannosyl glycolipids that are capable of stimulating iNKT cells have been identified to date, except for phosphatidylinositol mannosides in which the inositol 2′- and 6′- hydroxyl groups are substituted with different numbers of α-mannosides^24^. However, no detailed analyses have been conducted to demonstrate the interaction between the invariant TCR and the α-mannosyl residues of the lipids. To the best of our knowledge, M-AcM-MAG and ChAcMan characterized in this study are the first examples of the activators for iNKT cells in which an α-mannose residue is likely to be involved in the recognition by the invariant TCR.

One of the most crucial requisites for being antigens for iNKT cells in the TCR-dominated activation model^25^ is that they are α-glycosylated lipids. On the other hand, β-GlcCer has been proposed one of the endogenous antigens for iNKT cells in the course of the cytokine-dominated activation model^26^. The signals received by invariant TCR accompanied by those from cytokine receptors are assumed to be necessary for the activation of iNKT cells in this model^25^. However, recent studies have indicated that the interaction of the β-GalCer /CD1d or β-GlcCer/CD1d complex with the murine invariant Vα14 TCR was undetectable and that the activation of iNKT cells after the stimulation with the preparation of β-anomeric glycolipids may be due to a contaminating trace α-anomeric glycolipids^27, 28^. Thus antigenicity of β-glycosylceramides for iNKT cells may remain uncertain.

The hydrogen bond between the Gal 4′- axial hydroxyl group of α-GalCer and the invariant TCR α chain has been suggested to contribute to the stabilization of the lipid antigen/CD1d/invariant TCR ternary complex^29, 30^. This stabilization may account for the superiority of α-galactosyl lipids to α-glucosyl or α-mannosyl lipids as iNKT cell antigens.

The inappropriate configuration of the 4′ hydroxyl group, as well as the 2′ hydroxyl group of the sugar residue as described in the Introduction, is a disadvantage to α-mannosyl glycolipids as antigens for iNKT cells. In fact, no antigenicity was detected from M-DAG, α-ManCer or M-GU-DAG in (Fig. 2). In contrast, M-AcM-MAG and ChAcMan were identified as stimulators for iNKT cells in the present study. These α-mannosyl glycolipids are composed of structural units in unique combination. One of the two long hydrophobic moieties, each assumed to be received by either the A′ or the F′ pocket of CD1d, is an acyl group directly linked to the 6′ primary alcohol of the sugar residue. Here, it has been known that modifications to the 6′- hydroxyl group of α-glycosyl glycolipids do not significantly influence their antigenicity^8^. The sugar moiety of these antigenic α-mannosyl glycolipids may be less flexible than that of the other antigenic glycolipids so far reported when they are presented by CD1d because of the direct anchoring of the sugar-bound acyl chain to the pocket of CD1d. Suppose that the sugar residue of these glycolipids is fixed to the conformations suitable for the recognition by the invariant TCR for relatively a longer period, they will become more antigenic for iNKT cells. Interestingly, EhPIb, recently isolated from *Entamoeba histolytica* trophozoites and shown to activate iNKT cells, has a similar structure to these α-mannosyl glycolipids^31^; the 2′-hydroxyl group of the *myo*-inositol residue (next to the phosphorylated hydroxyl group) is acylated.

Immune responses by iNKT cells to M-AcM-MAG and ChAcMan were less intensive than those to α-GalCer (Fig. 2). The dose dependency of the responses by iNKT cells indicates that a higher dose (5~10 μg/ml) is required for these glycolipids to attain the maximal induction of the responses than the dose for α-GalCer (less than 0.5 μg/ml) (Supplemental Information Figure S1). Thus, the affinity of invariant Vα14 TCR to these α-mannosyl glycolipids appears to be weaker than that to α-GalCer. Staining profiles with the fluorescence-labeled CD1 tetramers/glycolipids complex shown in Fig. 3A support this speculation. Similarly, other microbial glycolipid antigens for iNKT cells so far reported appear to have somewhat less affinity to invariant Vα14 TCR than α-GalCer^8^. In addition to the inappropriate sugar residue (α-mannoside instead of α-galactoside) of the glycplipids, less stable anchoring of them to CD1d may also be another reason for the decreased affinity of the invariant TCR for the sugar residue of them compared with that of α-GalCer. This speculation is supported by the observation that the immune responses of iNKT cells induced with cholesteryl acyl α-galactoside (ChAcGal) were weaker than with α-GalCer (Supplemental Information Figure S2).

M-AcM-MAG is expressed by *Saccharopolyspora* ^11^, which is responsible for farmer’s lung disease^12^. iNKT cells were recently suggested to be involved in the prevention of hypersensitivity pneumonitis during farmer’s lung disease^32, 33^. It is possible that the recognition of the α-mannosyl glycolipid by iNKT cells triggers the immune responses to this microorganism.

ChAcMan was previously reported to be produced by *Candida albicans* ^15, 16^, responsible for various candidiasis. Cholesteryl 6-acyl glucoside (ChAcGlc) is also a natural product of bacteria^34^. *Helicobacter pylori* extracts cholesterol from the epithelial cells of the host and converts it to ChAcGlc^35^. We have recently demonstrated that ChAcGlc induces immune responses from iNKT cells in a CD1d-dependent manner^10^. Similir to ChAcMan and ChAcGlc, synthetic cholesteryl 6-acyl galactoside (ChAcGal) was also stimulative to iNKT cells (Supplemental information Fig. 2). These findings indicate that cholesteryl α-glycosides, as well as α-glycosyl ceramides and DAGs, are potent antigens for iNKT cells. The intensities of the immune responses induced by these cholesteryl acyl glycosides were on a similar level based on the induction of cytokine production by iNKT cells. Interestingly, superiority of α-galactosides as antigenic glycolipids for iNKT cells was conserved in a series of cholesteryl glycosides (without acyl group at the 6′-O-hydroxyl group); cholesteryl galactoside but not cholesteryl mannoside or glucoside was stimulus.

ChAcMan induced the IFN-γ-dominated cytokine production, even from CD1^−/−^ liver MNCs (Fig. 2). This CD1d-independent IFN-γ production may be triggered by the recognition of ChAcMan by cells other than iNKT cells. Wild-type splenocytes produced TNF-α and IL-6 following the stimulation with ChAcMan, and this production was abrogated by the depletion of Mac-1^+^ cells from splenocytes before cultivation (Fig. 7A). Splenocytes isolated from the *nu/nu* strain did not produce IFN-γ in response to the stimulation with ChAcMan or M-AcM-MAG. Thus, activated Mac-1^+^ cells may contribute to the activation of any T-lineage cells in addition to iNKT cells. These results suggest that any PRRs, presumably carbohydrate recognizing C-type lectin receptors, expressed by Mac-1^+^ cells are responsible for the recognition of ChAcMan. In fact, bone marrow-derived macrophages produced inflammatory cytokines such as TNF-α and IL-6 after being cultured in the presence of this glycolipid (Fig. 7B).

Interestingly, splenocytes, liver MNCs or bone marrow-derived macrophages produced only slight amount of IL-12 in response to the stimulation with ChAcMan or M-AcM-MAG (Shimamura, M. *et al*., unpublished results). This finding is in sharp contrast to the previous reports indicating that iNKT cells are activated in cytokine-dominated activation model with IL-12 secreted by antigen-presenting cells that are able to detect fungal or bacterial antigenic glycans by TLRs^25^, Dec-1^36^ or Dec-2^37^. A recent study reported that the fungal engagement of C-type lectin Mincle suppressed Dectin-1-induced antifungal IL-12 production^38^; therefore, C-type lectin receptors such as Mincle expressed by Mac-1^+^ cells may be candidate receptors for the glycolipids.

The physiological implications of the potential cross-priming of invariant Vα14 TCR and any innate immune receptors with certain microbial α-mannosyl glycolipids currently remains unclear. Individual mechanisms for recognizing foreign antigens may allow the host to effectively amplify the immune responses to infection. The α-mannosyl glycolipids are capable of inducing activation of iNKT cells in a manner independent of the engagement of any innate PRRs, while CD1d-independent IFN-γ production found in the culture of liver MNCs following the stimulation with ChAcMan (Fig. 2) is possibly initiated by the recognition of the lipid by PRRs. Interestingly, the CD1-independent IFN-γ production was not observed in the presence of ChAcGlc or ChAcGal (Supplemental information Figure S2). Thus, the α-mannosyl residue of ChAcMan is indispensable for the stimulation of putative PRRs. In addition, cytokine production induced by the engagement of innate PRRs with the α-mannosyl lipids may partially contribute to the initiation of iNKT cell activation via the cytokine-dominated activation mechanism. In fact, partial decrease in the activation of iNKT cells was observed in the presence of the antibody against one of the PRRs when they were co-cultured with antigen-presenting cells previously loaded with the α-mannosyl glycolipids (Shimamura *et al*., unpublished results). However, the results that the prompt IL-4 production by liver MNCs following stimulation with M-AcM-MAG or ChAcMan *in vitro* (Fig. 2) and *in vivo* (data not shown), and that the activation of iNKT cell hybridomas (Fig. 3B) and clones (Fig. 6B) with the α-mannosyl glycolipids presented by immobilized CD1d proteins suggest that iNKT cells were primarily stimulated via the direct TCR-dominated rather than the cytokine-dominated activation model. Further examinations are necessary to determine the extent of the two pathways of iNKT cell activation with these α-mannosyl glycolipids.

Protection of mice against infection with *S*. *pneumoniae* was demonstrated by administration of ChAcMan even when given 12 h after infection, thus suggesting the therapeutic potential of this glycolipid (unpublished results by P. Andrew *et al*.). Involvement of IFN-γ secreted from iNKT cells in the prevention of infection against *S*. *pneumoniae* was reported^22, 23^. Thus, administration of ChAcMan presumably enhanced the immune responses in mice to the infection via the activation of iNKT cells. Production of ChAcMan by *S*. *pneumoniae* remains uncertain, but it is likely that some α-mannosyl glycolipids corresponding to ChAcMan are expressed by the bacterium and recognized by invariant NKT cell receptor as well as by certain PRRs and become targets of the host immune system. The demonstration of new type of glycolipid antigens for iNKT cells suggests the roles of iNKT cells for the defense against wider range of microbial infection.

## Materials and Methods

### Mice

C57BL/6, BALB/c and BALB/c (*nu/nu*) mice were purchased from Sankyo Service Co. (Tokyo, Japan). CD1-deficient mice were provided by Dr. M. J. Grusby (Harvard University)^39^. They were backcrossed with C57BL/6 mice for 6 generations, and mice with the H-2^b^, NK1.1^+^ and CD1^−/−^ phenotypes were selected. MF1 outbred mice were purchased from Charles River (Margate, UK).

The animal experiments performed at Mitsubishi Kagaku Institute of Life Sciences were in strict accordance with the Japanese animal welfare bodies (Law No. 105 dated 19 October 1973 modified on 2 June 2006), and the plans of the experiments were reviewed and approved by the Ethics Committee of Mitsubishi Kagaku Institute of Life Sciences. The animal experiments performed at the University of Leicester were approved by the U. K. Home Office and the University of Leicester (Licence no. 60/4327 and 80/10279). Every effort was made to minimize suffering. In bacterial infection experiments mice were humanely culled if they became lethargic.

### Cell preparations

Mononuclear cells (MNCs) were prepared from single cell suspensions of mouse organs by density gradient centrifugation using Lymphosepar II (IBL, Gunma, Japan, *d* = 1.090) for the spleen and bone marrow and Percoll (Pharmacia, BD Biosciences, Uppsala, Sweden) for the liver as described previously^40^.

### Stimulation of lymphocytes with bacterial glycolipids in cultures

MNCs were isolated from the livers or spleens of mice (8–12 weeks of age). They were cultured at a concentration of 10^6^/200 μl DMEM (including 10% v/v FCS, 50 μg/ml streptomycin, and 50 U/ml penicillin) with the addition of the indicated glycolipids dissolved in DMSO or PBS containing 0.1% v/v Tween 20. Culture supernatants were analyzed for cytokines by ELISA. In some experiments, cells were cultured in the presence of anti-CD1 antibodies (clone 1B1, BD Biosciences, San Jose, CA, USA, 10 μg/ml) or the isotype matched rat IgG2b antibodies. In other cases the MNCs of splenocytes prepared from BALB/c and BALB/c (*nu/nu*) mice were treated with biotinylated anti-Mac-1 antibodies (M1/70.15, Cedarlane, Canada) followed by streptavidin-coated magnetic beads (Dynabeads, Veritas, Japan) to remove Mac-1^+^ cells according to the manufacturers’ protocols. Total or Mac-1^+^-depleted splenocytes were cultured in the presence of glycolipids. After 2 days inflammatory cytokines secreted into the supernatants were measured by ELISA.

### Stimulation of invariant Vα14 TCR^+^ cell hybridomas

The bone marrow cells of C57BL/6 and CD1^−/−^ mice were cultured in the presence of GM-CSF (2 ng/ml, PeproTech, London, UK) for 5 d, and the suspended cells were used as dendritic cells^41^. They were incubated with glycolipids for 16 h. After washing with DMEM, bone marrow cells (1 × 10^6^) were irradiated (3000 R) and cultured with invariant Vα14 NKT cell hybridomas^20^ (1 × 10^5^) in 200 μl DMEM for 1 day. IL-2 secreted into the culture supernatants was measured by ELISA.

In other experiments, 96-well plastic culture plates were pre-coated with CD1d/β2 m dimers (recombinant soluble dimeric mouse CD1d:Ig fusion protein, BD Biosciences, 10 μg/ml dissolved in PBS). After being washed twice with PBS, glycolipids dissolved in PBS containing tween 20 were added to the wells and incubated at room temperature for 1 day. The wells were washed twice with DMEM. Invariant Vα14 NKT cell hybridomas (1 × 10^5^) in 200 μl DMEM were added to the wells and cultured for 2 days. IL-2 secreted into the culture supernatants was measured by ELISA.

### Culture of bone marrow derived macrophages

The bone marrow cells of C57BL/6 mice were cultured in the presence of GM-CSF (2 ng/ml, PeproTech, London, UK) for 5 days. The suspended cells were removed and cells attached to the culture plate were detached with PBS including 0.05 M EDTA. After washing with DMEM, they were incubated with glycolipids for 2 days. Inflammatory cytokines secreted into the culture supernatants was dmeasured by ELISA.

### Culture of human Vα24 NKT cell clone cells in the presence of α-mannosyl glycolipids

CD1d-restricted human NKT cell clone BM2a.3 and CD1d-transfected K562 cells were established as previously described^42^. BM2a3 cells were stimulated with CD1d-transfected K562 cells previously loaded with M-AcM-MAG or ChAcMan in the presence or absence of anti-CD1d antibodies (clone 12.1.1.1, Pharmingen, BD Bioscience) as previously described^43^, and the immune responses were assessed by measuring IFN-γ production.

In other experiments BM2a3 cells were stimulated with plate-immobilized human CD1d proteins. Nunc MaxiSorp 96-wells plates (eBioscience, USA) were coated with recombinant human CD1d/β2 microglobulin Fc fusion proteins (0.5 µg/well; kindly provided by Dr. Jenny Gumperz^44^) and anti-LFA-1 antibodies (0.05 µg/well, AbD Serotec) dissolved in PBS for overnight at 4 °C. After washing with PBS, the immobilized CD1d proteins were loaded with sonicated lipid antigens diluted in 25% v/v DMSO in distilled water for overnight at 37 °C. After washing with PBS and then DMEM, BM2a3 cells were added to the well (5 × 10^4^ cells/well). They were cultured for overnight at 37 °C and the culture supernatants were analyzed for IFN-γ by ELISA.

### Flow cytometry and antibodies

Liver MNCs from mice were pre-treated with anti-FcγRII, III monoclonal antibodies (2.4G2, Pharmingen, BD Biosciences), to saturate Fc receptors. Specific staining was performed with a combination of Tricolor-labeled anti-TCR Cβ antibodies (H57-597, Pharmingen, BD Biosciences), complexes of the PE-labeled CD1d tetramers and glycolipids and FITC-labeled anti-CD69 antibodies (Pharmingen, BD Biosciences). CD1 tetramer proteins were purchased from MBL Life Science (Nagoya Japan). Stained cells were analyzed on a FACScan flow cytometer equipped with the Cell Quest Software (BD Biosciences).

### Glycolipids

ChAcGlc (PI-57) was synthesized as described previously^10^. α-GalCer and α-ManCer were prepared according to previously described protocols^45^. M-AcM-MAG (PI-97) was isolated from *Saccharopolyspora rectivirgula* as described^11^. M-DAG (PI-116) was prepared from *Streptococcus suis* as previously described^7^. ChAcMan (PI-89) and M-GU-DAG (PI-121) were synthesized in the laboratory of P. I.

Details of the preparation of M-AcM-MAG and ChAcMan are shown in Supplemental Information.

### *In vivo* evaluation of α-mannosyl glycolipid activity

Infection of mice with *S*. *pneumoniae* was performed as previously described^46, 47^. Ten-week-old female MF1 outbred mice (Charles River, Margate, UK) were lightly anesthetized with 3% (v/v) isoflurane over oxygen, and administered intranasally with an inoculum of 50 μl containing approximately 1 × 10^6^ CFU in PBS (pH 7.0). ChAcMan or M-AcM-MAG was dissolved at the concentration of 500 µg/ml in PBS containing 0.2% (v/v) Tween 20, heated to 80 °C for 10 min, and sonicated for 5 min before use^48^. Glycolipid (5 μg each) was then injected intraperitoneally in 100 μl sterile PBS either 30 min, or 12 h after infection. Mice were monitored for disease signs (progressively starry coat, hunched, and lethargic) for 7 days, and they were evaluated independently by two experienced operators. To quantify the disease signs, a score of 2, 4 or 6 was given if the mouse was hunched, had a starry coat, or was lethargic, respectively. When mice were lethargic, they were culled. Therefore, the time to reach the lethargic state has been defined as the “survival time.” Mice that were alive 7 days after infection were deemed to have survived the infection. Survival times were calculated using GraphPad Prism software and analyzed by the Mann-Whitney U test. To monitor the development of bacteremia in each mouse, approximately 20 μl venous blood was obtained from mice at pre-determined time points after infection. Viable counts in blood were determined by serial dilution in sterile PBS and plating onto blood agar plates supplemented with 5% (v/v) defibrinated horse blood. Bacterial counts in blood were analyzed by an analysis of variance followed by the Bonferroni post-test.

### ELISA analysis

Cytokines were measured by ELISA using anti-cytokine antibodies purchased from BD Biosciences.

### Statistical analysis

Data are shown as the mean ± s.d. (standard deviation). The significance of differences was determined by the Student’s *t*-test, the Mann-Whitney U test, or the Bonferroni post-test. Statistical significance was considered to be a *p* value of < 0.05.

## Electronic supplementary material

Supplemental Information
