# Supplementary material for: Activation of invariant natural killer T cells stimulated with microbial α-mannosyl glycolipids
Source: Sci Rep. 2017 Aug 29;7:9703. doi: 10.1038/s41598-017-10309-x (PMC5574887; doi:10.1038/s41598-017-10309-x)
Supplement: Supplementary file 1 — Supplemental Information [file 41598_2017_10309_MOESM1_ESM.doc]

**Supplementary Information**

**Activation of invariant natural killer T cells stimulated with microbial α-mannosyl glycolipids**

Michio Shimamura, Masaki Yamamura, Tatsuya Nabeshima, Naoki Kitano, Peter van den Elzen, Hasan Yesilkaya, Peter Andrew and Petr Illarionov

**Preparation of M-AcM-MAG (PI-97)**

M-AcM-MAG was isolated from *Saccharopolyspora rectivirgula* (strain IMRU1258) as described^1^. The mixture of CHCl_3_/CH_3_OH (2:1, v/v) was added to the *Saccharopolyspola* pellets and the suspension was stirred for 6 h. Supernatant was removed and the pellets were re-extracted twice with CHCl_3_/CH_3_OH (2:1, v/v). The combined extracts were concentrated and the lipids were re-extracted by Folch method. The purification of M-AcM-MAG from the extracts in the Folch upper phase was achieved by silica gel (Fluka, 60 mesh) column chromatography using stepwise gradient of CHCl_3_/CH_3_OH (40:1to 4:1, v/v). The lipid extract was examined by TLC on aluminum-backed plates of silica gel 60 F254 (Merck 5554), using CHCl_3_/CH_3_OH,/H_2_O (65:25:4, v/v/v) ad developing solvent. Glycolipids were visualized by spraying plates with-naphthol/sulfuric acid followed by gentle charring of plates. The final preparation gave a single combined spot on the TLC plate (Rf. 0.5) mainly consisting of M-Ac-MAG of C17:0, C17:0 fatty acids.

The purified lipid was characterized by ^1^H-NMR spectroscopy and mass spectrometry analyses (Supplemental Figure S3). The obtained spectra were well in accord with those shown in the literature^1^.

**Synthesis of ChAcMan (PI-89)**

Cholesteryl mannoside was synthesized by glycosilation of cholesterol using 2’,3’,4’,6’-benzoyl–α-D-mannopyranosyl trichloroacetimidate as a sugar donor by the methods of Wu and Kong^2^ to obtain the α-anomer. After glycosylation, benzoyl groups were deprotected by treating the product with sodium methoxide.

ChAcMan was synthesized by selective acylation of the 6’-hydroxyl group of cholesteryl mannoside with tetradecanoyl chloride. ChAcMan was purified by silica gel chromatography (Sigma) using chloroform/ methanol (15:1) as eluent. The purity was checked by thin-layer chromatography. ^1^H-NMR spectrum and ESI-MS spectrogram of ChAcMan are shown in Supplemental Figure S4 and S5.

**^1^H-NMR analysis**. Deuterated solvents were purchased from Aldrich. ^1^H-NMR spectra of glycolipids were recorded in CDCl_3_ / CD_3_OD (2:1, v/v) at 300 K on a Bruker DRX300 operating at 300.13 MHz. Data were acquired and processed using XWINNMR version 2.6 software on a Silicon Graphics work station.

**Mass spectrometric analysis**. Glycolipids dissolved in　DMSO were diluted with CH_3_CN, filtrated and applied to Electrospray ionization mass spectrometric analysis in a positive mode (AB SCIEX Triple TOF4600).

**References**

1. Gamian, A. *et al.* Structural studies of the major glycolipid from *Saccharopolyspora* genus*.*  *Carbohydr. Res*. ***296, 55-67 (1996).***
2. Wu, Z. and Kong, F. Synthesis of mannose-containing analogues of (1-6)-branched (1-3)- glucohexose. *Carbohydr. Res*. ***339, 377-384 (2004).***

**Supplementary figures**

**
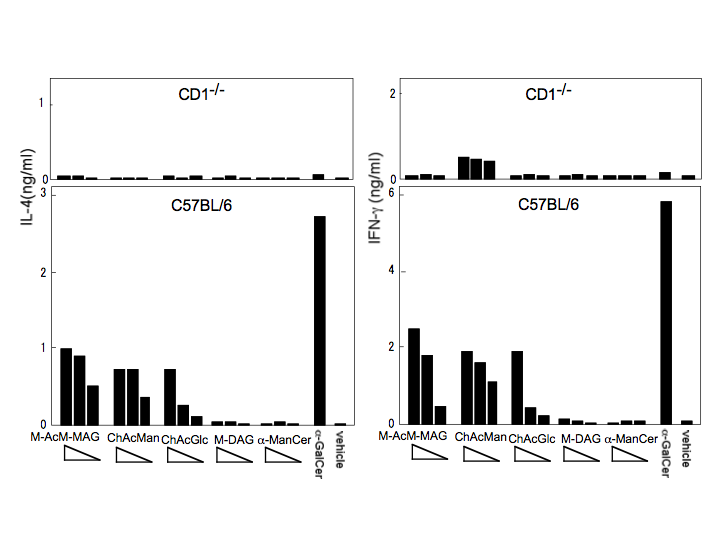
**

**Figure S1 Dose-dependent activation of iNKT cells with the α-mannosyl glycolipids**

Liver MNCs prepared from C57BL/6 and CD1^-/-^ mice (10^6^ /well) were cultured in the presence of the indicated α-mannosyl glycolipids, ChAcGlc (10 μg/ml, 5 μg/ml, 1 μg/ml) or α-GalCer (0.5 μg/ml). Cytokine concentrations in the culture supernatants were measured after 2 days by ELISA. The mean values of the triplicate cultures are shown.


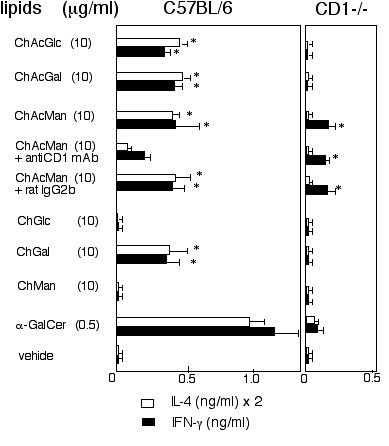


**Figure S2** **Immunostimulatory activity of cholesteryl glycoside derivatives to invariant Vα14 NKT cells.**

Liver MNCs prepared from C57BL/6 and CD1^-/-^ mice were cultured for 1 day in the presence of chemically synthesized cholesteryl 6’-O-acyl α-glycosides (10μg/ml), cholesteryl α-glycosides (10μg/ml) or α-GalCer (0.5 μg/ml). The immune responses were assessed by analyzing the cytokine concentrations in the culture supernatants. In some experiments anti-CD1 mAb or isotype-matched rat IgG2b (10 μg/ml) was added to the culture to evaluate the CD1-dependency of the responses. The mean values of the triplicate cultures are shown. Experiments were repeated 3 times, and one of the results giving similar cytokine profiles is shown. *, *p*<0.01in the Student’s *t*-test compared with the control (in the presence of vehicle).

**Figure S3 ESI-Mass spectrogram of M-AcM-MAG**

Positive mode ESI-Mass spectrograms of M-AcM-MAG (PI-97) are shown. (Left panel) The peak at m/z 943.6 corresponds to [M+Na]^+^ (C17:0, C17:0). Note that the weak peaks at m/e 929.6 and 915.6 correspond to [M+Na]^+^ (C16:0, C17:0) and [M+Na]^+^ (C16:0, C16:0), respectively. (Right panel) Isopope composition of [M+Na]^+^ (C17:0, C17:0). The found peaks are compared with the peaks calculated for C_49_H_92_O_15_Na.

**
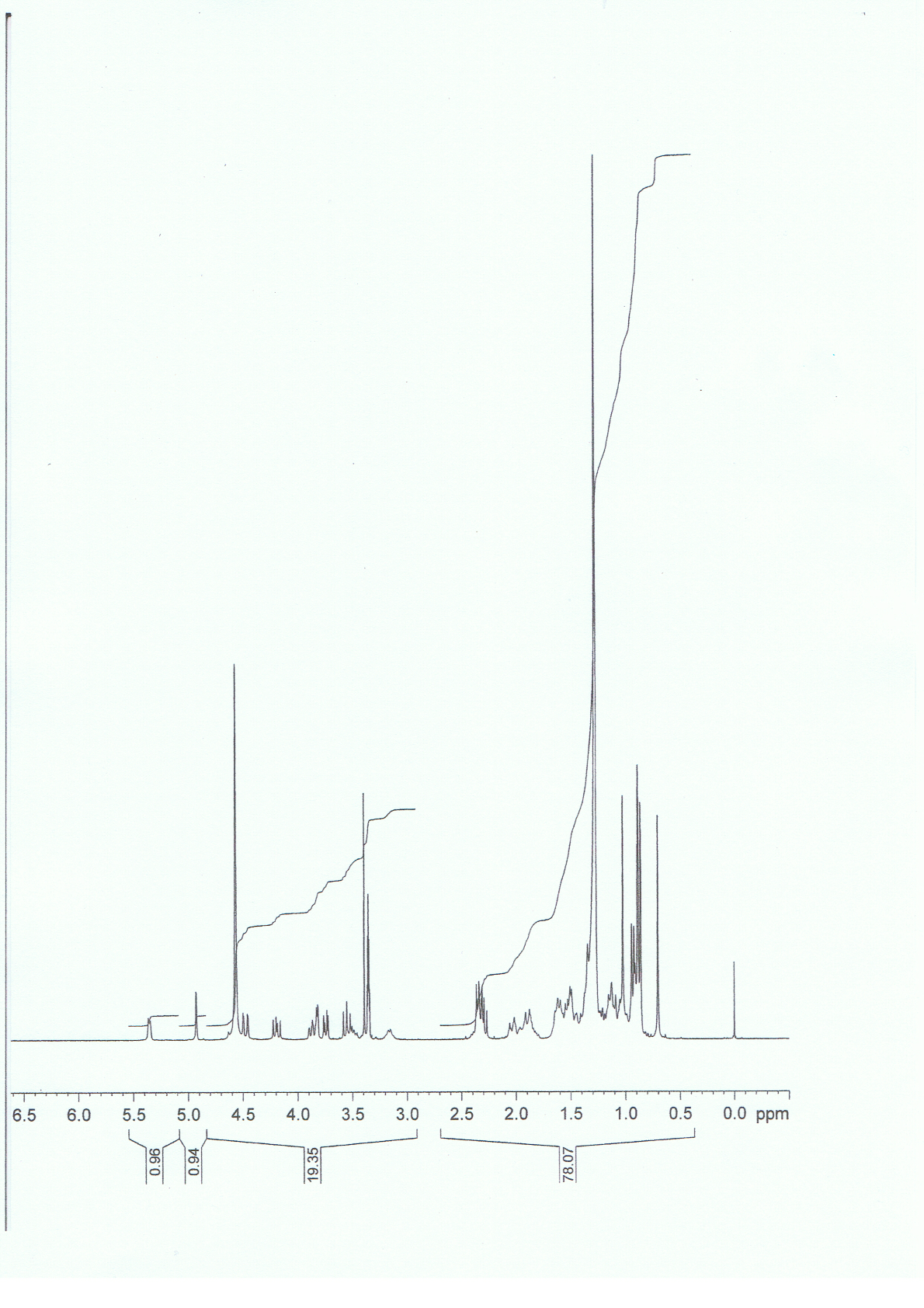
**

**Figure S4 ^1^H-NMR spectrum of ChAcMan**

^1^H-NMR spectrum of ChAcMan (Brucker DRX300, 300MHz, CDCl_3_/CD_3_OD, 2:1) δ 5.35 (t, *J*=3.0 Hz, 1H, C=CH-CH_2_, cholesterol), δ 4.93 (d, *J*=1.9 Hz, 1H, H-1 of α-mannoside), δ 4.47(dt, 1H, H-6a), δ 4.21 (dd, 1H, H-6b).

**Figure S5 ESI-Mass spectrogram of ChAcMan**

Positive mode ESI-Mass spectrograms of ChAcMan (PI-89) are shown. (Left panel) The peak at m/z 781.59 corresponds to [M+Na]^+^ (C14:0). (Right panel) Isopope composition of [M+Na]^+^ (C14:0). The found peaks are compared with the peaks calculated for C_47_H_82_O_7_Na.
